# Laparoscopic Transcystic Common Bile Duct Exploration for the Treatment of Choledocholithiasis Following Roux-en-Y Gastric Bypass

**DOI:** 10.1007/s11695-026-08688-0

**Published:** 2026-05-14

**Authors:** Eric D. Moyer, McKell Quattrone, Andrew J. Rothka, Elizabeth Sodomin, Eric M. Pauli, Joshua S. Winder

**Affiliations:** 1 Division of Minimally Invasive and Bariatric Surgery, Penn State Milton S. Hershey Medical Center, Hershey, USA; 2 Department of Surgery, University of North Carolina at Chapel Hill, Chapel Hill, USA

**Keywords:** Choledocholithiasis, Gastric bypass, Choledochoscopy, Transcystic common bile duct exploration

## Abstract

**Introduction:**

Choledocholithiasis is challenging to treat in patients who have undergone Roux-en-Y gastric bypass (RYGB). Laparoscopic transcystic common bile duct exploration (LTCBDE) performed at the time of the cholecystectomy is an alternative treatment modality that has the potential to reduce hospital length of stay (LOS) and postoperative morbidity.

**Methods:**

This is an IRB-approved, single-academic institution retrospective cohort study analyzing electronic medical records of patients with RYGB anatomy who underwent LTCBDE for choledocholithiasis between 2021 and 2025. The primary outcome of interest was procedural response. Secondary outcomes of interest included technical response rates, operative and 30-day postoperative complications. Hospital and postoperative LOS were compared between admitting services.

**Results:**

Nineteen patients with RYGB anatomy underwent LTCBDE and concomitant cholecystectomy. Technical response was achieved in 100% of patients, and procedural response was achieved in 94.7% of patients. One (5.3%) required an additional procedure after a retained stone was found incidentally on cross-sectional imaging for oncologic surveillance purposes. No intraoperative or postoperative complications were experienced. Patients admitted to a surgical service had a shorter hospital LOS by 3.1 days on average (*P* < 0.001; 95% CI -1.2,-2.6) and median postoperative LOS by 0.3 days (*P* = 0.035; 95% CI -0.1,-1.6) than those admitted to a medicine service.

**Conclusions:**

Our data demonstrate high rates of technical and procedural response with no complications, and a shorter overall hospital LOS when managed by a surgical team, supporting the use of LTCBDE to treat choledocholithiasis in patients with RYGB anatomy.

## Key points

LTCBDE is a safe and effective method to treat choledocholithiasis in patients with RYGB anatomy.

Patients who require LTCBDE experience a shorter hospital and postoperative LOS when admitted to a surgical service compared to a medicine service.

## Introduction

Approximately 30% of patients who undergo bariatric surgery will develop cholelithiasis within 12 to 18 months following their surgery [1–3]. Furthermore, 10–12% of patients who develop symptomatic cholelithiasis may progress to choledocholithiasis [4, 5]. Treatment of choledocholithiasis in patients with altered foregut anatomy, particularly patients who have undergone Roux-en-Y gastric bypass (RYGB), is challenging due to the problematic nature of performing a trans-oral endoscopic retrograde cholangiopancreatography (ERCP). Often, more complex procedures are needed to access the biliary system, such as laparoscopic-assisted transgastric ERCP (TG-ERCP), single- or double-balloon enteroscopy, or endoscopic ultrasound-directed Transgastric ERCP (EDGE). Due to the technical challenges associated with these procedures, they are typically limited to specialized centers and experienced clinicians.

Laparoscopic transcystic common bile duct exploration (LTCBDE), first described by Reddick et al. in 1990 [6], utilizes a small-caliber flexible endoscope to access the common bile duct (CBD) through the cystic duct. Direct visualization of the CBD allows for the identification and extraction of stones using retrieval baskets or snares. In situations where stones are too large to be extracted in their entirety, electrohydraulic lithotripsy (EHL) can be used to fragment the stones, making them easier to extract. Furthermore, LTCBDE can be performed concomitantly during cholecystectomy, thereby reducing hospital length of stay and associated costs.

Multiple previous studies have compared the safety and efficacy of LTCBDE to a transductal (choledochotomy) approach. A systematic review and meta-analysis found that both methods have similar response rates. However, the transcystic route was associated with a decrease in overall postoperative complications and biliary complications, a decreased hospital length of stay (LOS), and lower intraoperative blood loss [7]. Few studies focus on LTCBDE in patients who have previously undergone RYGB [8, 9]. The study aims to report our institutional experience with LTCBDE for the treatment of choledocholithiasis after RYGB.

## Methods

After receiving institutional review board approval, we retrospectively analyzed medical record data from a single academic institution between January 2021 and May 2025 of adult patients with a history of RYGB who underwent laparoscopic cholecystectomy with concomitant LTCBDE for definitive treatment of choledocholithiasis. We collected data related to basic demographics and associated medical conditions. Our primary outcome was procedural response, defined as the successful clearance of the biliary system of stones or debris, ensuring that no additional procedures are required (i.e., a retained stone requiring return to the operating room). Our secondary outcome measures included technical response (defined as the ability to successfully navigate the endoscope through the cystic duct into the CBD and visualize the entire distal CBD), operative time, adjuncts to LTCBDE, intraoperative and 30-day postoperative complications, total hospital and postoperative LOS.

Patient follow-up consisted of either post-procedural clinic visits, which focused on the previous biliary procedure, or telephone follow-up. Telephone follow-up was performed for any patient who had been more than three months since their most recent postoperative clinic visit. The telephone follow-up focused on specific questions aimed at assessing the patient for any recurrence of symptoms such as right upper quadrant or epigastric pain, nausea or emesis, particularly after eating fatty foods, or jaundice. Additionally, patients were asked if they required any additional biliary interventions after their initial LTCBDE.

Description of the LTCBDE technique.

**Fig. 1:**
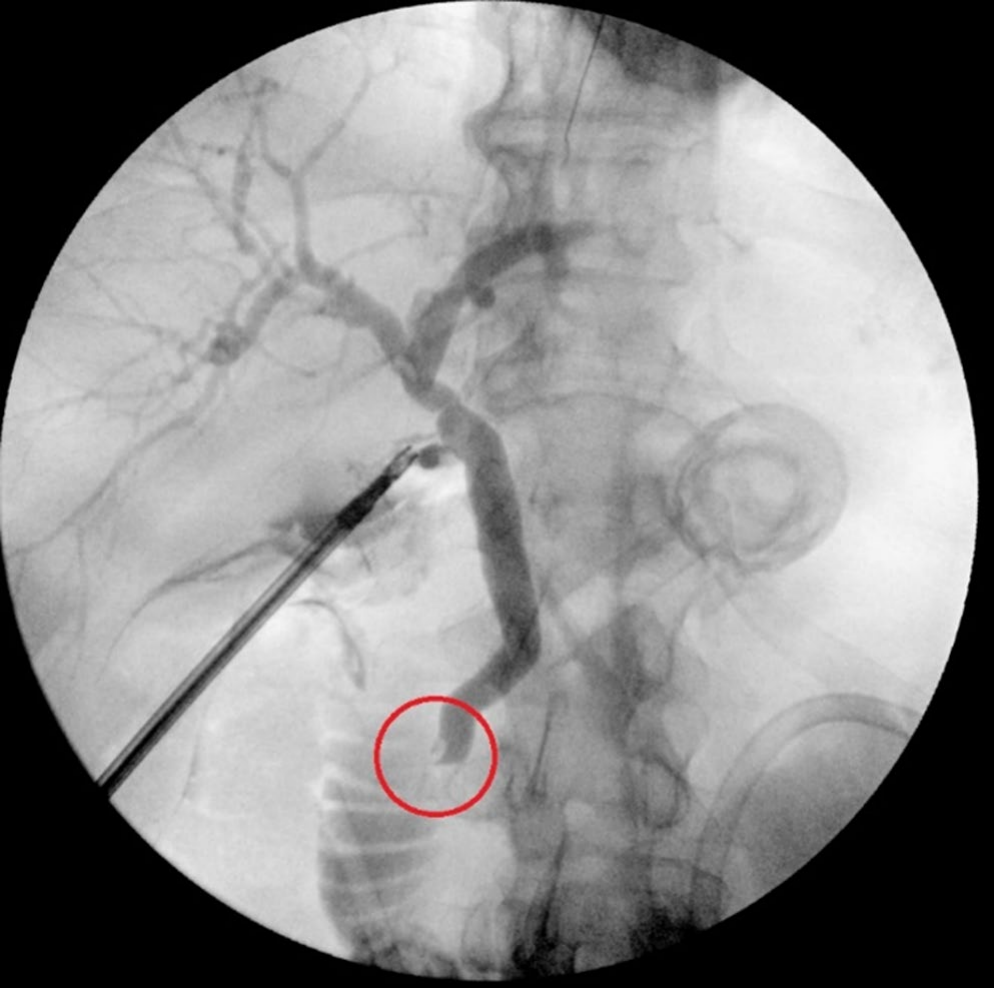
Intraoperative cholangiogram demonstrating a filling defect in the distal common bile duct

LTCBDE is a procedure that can be performed during a laparoscopic cholecystectomy regardless of foregut anatomy to access the CBD to treat choledocholithiasis. Indications for LTCBDE include absolute or equivocal filling defects within the CBD on intraoperative cholangiography, CBD stones smaller than 10 mm in diameter, fewer than nine total stones, and evaluation of CBD strictures (both benign and malignant) [10]. Contraindications to this method include a friable cystic duct (which we would classify as an absolute contraindication), an anatomically small cystic duct, stones larger than one cm, and ten or more stones [10]. Once the critical view of safety is obtained, the cystic artery may be clipped and divided. A single clip is placed on the cystic duct close to the infundibulum to give as much working area as possible for a cystic ductotomy. A transverse ductotomy is made utilizing laparoscopic scissors close to the clip on the distal cystic duct. An intraoperative cholangiogram is performed in the standard fashion using a 5Fr cholangiogram catheter (Fig. 1).

**Fig. 2:**
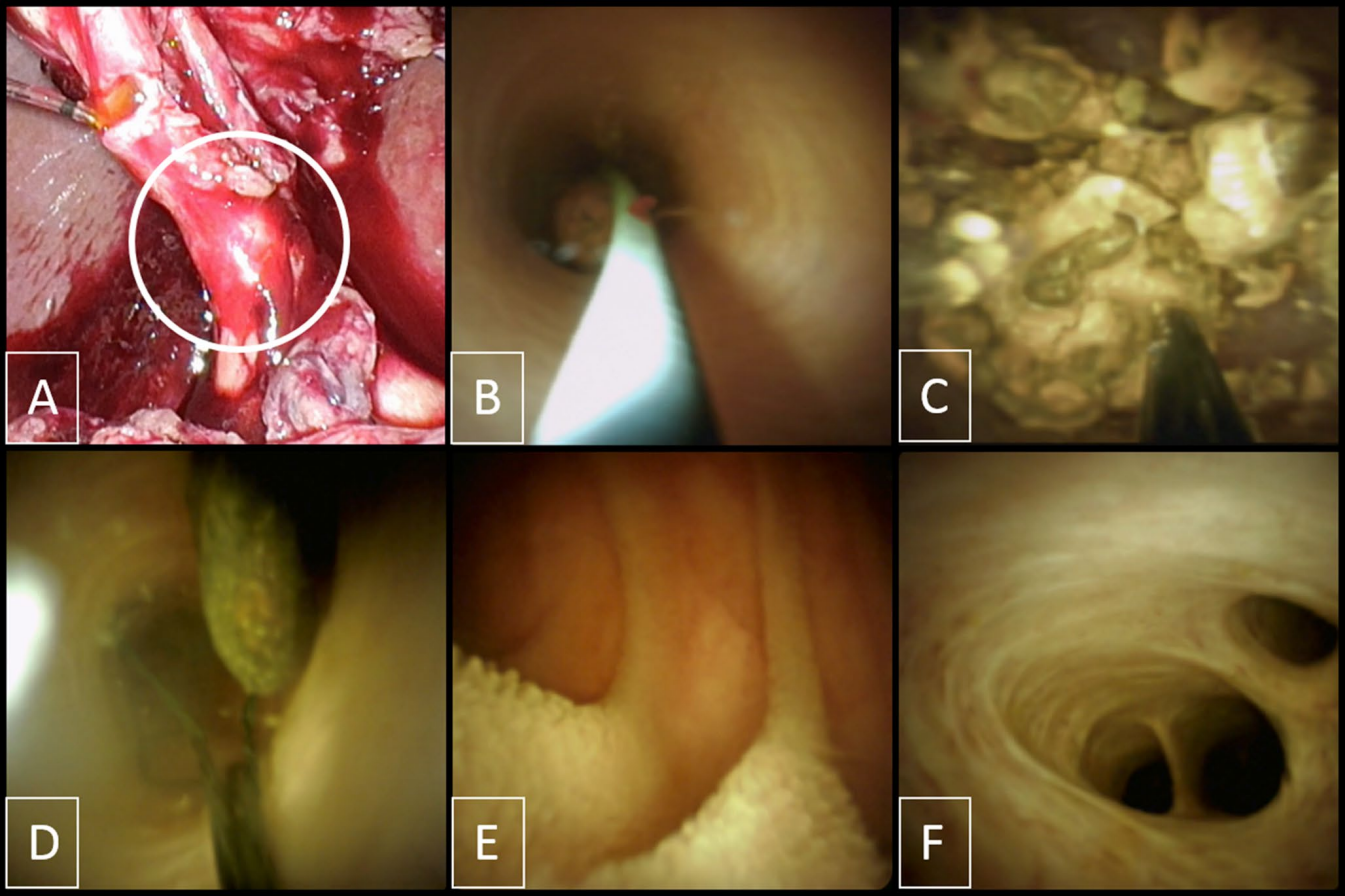
Key steps in LTCBDE. **A** Balloon dilation of the cystic duct. **B** Advancement of the flexible choledochoscope over a wire into the CBD (floating calculus visible within the distal CBD). **C** Fracturing of distal CBD calculus using electrohydraulic lithotripsy. **D** Basket extraction of free-floating CBD calculus. **E** Transampullary view of duodenal mucosa. **F** Retrograde view of the common hepatic duct without evidence of filling defects

The operating surgeon interprets the intraoperative cholangiogram, which is crucial in determining the location of any obstructing or free-floating stones within the biliary tree. A flexible wire is passed through the cholangiogram catheter, ensuring it travels to the distal CBD via fluoroscopy. An 8 mm balloon-dilating catheter is passed over the wire through the ductotomy to dilate the cystic duct to accommodate a choledochoscope (Fig. 2A). Then, over the wire, a 10Fr therapeutic flexible choledochoscope is passed through the ductotomy and into the CBD with intermittent saline irrigation. Avoiding excessive traction on the cystic duct to prevent duct avulsion or injury to the CBD is essential. Once in the CBD, any obstructing or free-floating stones visible can be removed using a retrieval basket (Fig. 2B and D). Additionally, electrohydraulic lithotripsy (EHL) probes can also be advanced through the working channel of the choledochoscope to fracture larger calculi that cannot be removed with a retrieval basket alone (Fig. 2C). Additional maneuvers, such as glucagon administration, can also be attempted to flush stones or fragments into the duodenum if necessary. Clearance of the proximal CBD is confirmed by the passage of the choledochoscope through the ampulla with visualization of duodenal mucosa (Fig. 2E). The choledochoscope is then withdrawn to the junction of the cystic duct and CBD and maneuvered proximally to view the common hepatic duct to identify any proximal stones (Fig. 2F). A completion cholangiogram is performed to confirm no remaining filling defects within the CBD. The choledochoscope is then removed from the cystic ductotomy. Two additional clips are placed on the proximal cystic duct, which is then transected, and the remainder of the cholecystectomy is completed.

### Data Analysis

Data was analyzed using Stata SE version 18.5 (College Station, Texas). Data normality was assessed using the Shapiro-Wilk test. Continuous parametric data is expressed as mean (± SD), while continuous non-parametric data is expressed as median (IQR). For continuous variables that did not follow a normal distribution, comparisons between two groups were conducted using the Wilcoxon rank-sum test. Confidence intervals for the Wilcoxon rank-sum test were estimated using the Hodges-Lehmann estimator to measure the median difference between groups. For continuous variables meeting normality assumptions, two groups were compared using a two-tailed t-test. Statistical significance was determined using a p-value threshold of 0.05.

## Results

### Demographic Data

Nineteen patients with RYGB anatomy underwent LTCBDE between January 2021 and May 2025. Table 1 illustrates the demographics and associated medical conditions of patients included in this study. The majority of the cohort was female (68.4%), with a mean age of 61.5 years (SD ± 9.6) and a mean BMI of 34.6 kg/m² (SD ± 10.9). Three (15.8%) patients were actively smoking at the time of their procedure.

**Table 1:** Demographics

		*N* = 19
Female		13 (68.4%)
Mean age, years (SD)		61.5 (± 9.6)
Race	White	18 (94.7%)
	Other	1 (5.3%)
Mean BMI, kg/m^2^ (SD)		34.6 (± 10.9)
ASA	2	10 (52.6%)
	3	9 (47.4%)
History of RYGB		19 (100%)
Smoking status	Never smoker	10 (52.6%)
	Current smoker	3 (15.8%)
	Quit greater than 1 year ago	6 (31.6%)
HTN		9 (47.4%)
Diabetes		1 (5.3%)
COPD		2 (10.5%)
A fib		1 (5.3%)

### Preoperative Data

Table 2 illustrates the preoperative data. Eleven (57.9%) patients were admitted to a medical service upon presentation to the hospital, with the remaining 8 (42.1%) admitted directly to a surgical service. Most patients had a preoperative diagnosis of choledocholithiasis (17, 89.5%). However, other diagnoses included cholangitis (3, 15.8%), cholecystitis (7, 36.8%), and symptomatic cholelithiasis (8, 42.1%). All patients underwent preoperative imaging, with the majority being MRCP (15, 78.9%), which demonstrated a median CBD diameter of 9 mm (IQR, 7–10 mm). The median size of CBD filling defects appreciated in patients with a preoperative diagnosis of choledocholithiasis was 5 mm (IQR 4–8 mm). Four cases (21.1%) were scheduled as elective procedures, 13 cases (68.4%) were non-emergent same-admission procedures, and two cases (10.5%) were classified as urgent.

**Table 2:** Preoperative data

		*N* = 19
US		3 (15.8%)
MRCP		15 (78.9%)
CT		1 (5.3%)
Imaging findings	n with CBD size	19 (100%)
	Median CBD size, mm (IQR)	9 (7–10)
	n with CBD stone size	16 (84.2%)
	Median CBD stone size, mm (IQR)	5 (4–8)
Indication for procedure	Cholangitis	3 (15.8%)
	Cholecystitis	7 (36.8%)
	Choledocholithiasis	17 (89.5%)
	Symptomatic cholelithiasis	8 (42.1%)
Patient acuity	Elective	4 (21.1%)
	Non-emergent	13 (68.4%)
	Urgent	2 (10.5%)
Admitting service	Medicine	11 (57.9%)
	Surgery	8 (42.1%)

### Operative Data

Table 3 illustrates the operative data collected. The mean operative time was 178 min (± 63 min). All patients underwent an intraoperative cholangiogram to confirm the need for LTCBDE. During the endoscopic exam, most patients (14, 73.7%) had stones visualized in the distal common bile duct, while three (15.8%) patients had stones in the hepatic duct, and three (15.8%) had a stone in the cystic duct. Five (26.3%) had no calculi or debris visualized on endoscopic exam. Single stones were encountered in three (15.8%) patients, two stones in three (15.8%) patients, and multiple stones in eight (42.1%) patients. Fourteen (73.7%) patients required adjuncts to clear their CBD, including glucagon (8, 42.1%), basket extraction (10, 52.6%), EHL (6, 31.6%), and balloon sphincteroplasty (3, 15.8%). One (5.3%) patient had preoperative imaging suggestive of a duodenal mass, which required placement of a laparoscopic gastrostomy tube into the remnant stomach to facilitate an endoscopic biopsy of the mass. This patient underwent LTCBDE before placement of the gastrostomy tube and did not require TG-ERCP. The gastrostomy tube remained in place for potential future surveillance; however, it was removed two months post-operatively in the outpatient clinic after the lesion was determined to be benign. We experienced a 100% technical response rate with no intraoperative complications. All patients had a completion cholangiogram demonstrating no residual filling defects after their LTCBDE.

**Table 3:** Operative Data

		*N* = 19
Technical response		19 (100%)
Mean operative time, min (SD)		178 (± 63)
Adjuncts	n requiring adjuncts	14 (73.7%)
	Electrohydraulic lithotripsy	6 (31.6%)
	Glucagon	8 (42.1%)
	Basket extraction	10 (52.6%)
	Balloon sphincteroplasty	3 (15.8%)
Number of stones	Zero	5 (26.3%)
	One	3 (15.8%)
	Two	3 (15.8%)
	Multiple	8 (42.1%)
Location of stones	Cystic duct	3 (15.8%)
	Common bile duct	13 (68.4%)
	Hepatic duct	3 (15.8%)
Hepatic duct visualized endoscopically		8 (42.1%)
Access to the remnant stomach		1 (5.3%)
Gastrostomy tube remained		1 (5.3%)
Operative complication		--

### Postoperative Data

Table 4 illustrates the postoperative data collected. The mean hospital LOS and median postoperative LOS were 3.4 days (± 2.1 days) and 1.2 days (IQR 1.0-1.9 days), respectively. Upon subgroup analysis, illustrated in Table 5, the mean hospital length of stay was 3.1 days shorter for patients admitted to a surgical service compared to those admitted to a medicine service (*P* < 0.001; 95% CI -1.2,-2.6). Similarly, the median postoperative LOS was 0.3 days shorter for patients admitted to a surgical service than those admitted to a medicine service (*P* = 0.035; 95% CI -0.1,- 1.6). All patients underwent follow-up, with a median time to follow-up of 195 days (IQR 43–188 days). We achieved a 94.7% procedural response rate (*n* = 18). One (5.3%) patient was found to have a retained CBD stone measuring 5 mm on an oncologic surveillance imaging study. Although the patient remained asymptomatic, because of its size and location within the distal CBD, they underwent percutaneous transhepatic biliary drain placement followed by endoscopic retrieval of the retained stone five months after their initial procedure. There were no postoperative adverse events reported in any of the 19 patients.

**Table 4:** Postoperative Data

		*N* = 19
Median preoperative LOS, days (IQR)		1.2 (0.5–2.9)
Median postoperative LOS, days (IQR)		1.2 (1-1.9)
Mean hospital LOS, days (SD)		3.4 (± 2.1)
Follow-up	n	19 (100%)
	Median length of follow-up, days (IQR)	195 (43–788)
Postoperative complications	Patients with postoperative complications	--
	Total number of complications	--
	Retained stones	1 (5.3%)
Procedural response		18 (94.7%)

**Table 5:** Subgroup analysis comparing length of stay based on admitting service

	Admitted to Surgical Service	Admitted to Medicine Service	*p*-value [95% CI]
Mean hospital LOS, days (SD)	1.6 (± 0.8)	4.7 (± 1.8)	< 0.001 [-1.2,-2.6]
Median postoperative LOS, days (IQR)	1.0 (0.8–1.5)	1.3 (1.1–2.9)	0.035 [-0.1,-1.6]

## Discussion

In this cohort study, we demonstrate the safety and efficacy of LTCBDE in treating choledocholithiasis in patients with RGYB anatomy. Few studies have focused on this procedure explicitly in patients with RYGB anatomy. As previously described, the altered foregut anatomy presents unique challenges to accessing the ampulla and cannulating the CBD. Most endoscopic procedures currently performed, such as single- or double-balloon enteroscopy, TG-ERCP, and the EDGE procedure, have their unique risk profiles and often result in multiple trips to the operating room or procedure suite, along with the associated anesthetic exposure. Additionally, they may require coordination of the various teams (surgical and gastroenterology) needed to perform these procedures, which presents a logistical challenge and likely contributes to increases in hospital LOS.

Our study reports high technical and procedural response rates of 100% and 94.7%, respectively, which are comparable to those in previously published studies. Although studies focusing on RYGB anatomy are quite limited in the literature, procedural response rates for this population range between 90.9% and 98% [8, 9]. For all-comers, procedural response rates are approximately 91% to 95% [7, 11]. Technical response rates are rarely explicitly reported in the literature; however, the available data suggest that they are around 94.4% to 100% [8, 12]. Table 6 illustrates the procedural and technical response rates of other commonly performed procedures for treating choledocholithiasis in RYGB anatomy.

**Table 6:** Comparison of treatment modalities for choledocholithiasis in RYGB anatomy

Procedure	Technical response	Procedural response	Postoperative adverse events
LTCBDE (%) [7–9, 12–14]	94.4–100	91-98.5	4.6–16.7
Single- or Double-Balloon Enteroscopy (%) [15–19]	59–89	56–85	3.1–11.1
EDGE (%) [17, 19–21]	89–100	85–100	8-27.8
TG-ERCP (%) [16, 19, 20, 22, 23]	94–100	94–100	8.3–12.8

Our single case of a retained CBD stone demonstrates a unique approach to accessing the CBD following cholecystectomy. This patient had a 5 mm filling defect within the common hepatic duct on the initial cholangiogram at the time of the LTCBDE, which could not be identified on endoscopic evaluation. It is our practice to retroflex into the common hepatic duct; however, the orientation of the cystic duct-CBD junction prohibited this maneuver in this patient. With the distal common bile duct devoid of stones, we performed a completion cholangiogram, which failed to reveal any remaining filling defects throughout the entire biliary system, so the procedure was concluded. Three months after the procedure, the patient underwent an MRI of the abdomen for oncologic surveillance, revealing a 5 mm retained stone within the distal CBD. Because of the patient’s previous cholecystectomy, access to the CBD via the transcystic route was not possible. We opted for an ultrasound-guided 10 French internal/external biliary drain to be placed across the ampulla, which allowed repeat endoscopic evaluation and stone retrieval through the drain tract four weeks after drain insertion. Aside from needing repeat procedures, the patient experienced no additional adverse sequelae.

As with any procedure, operative complications are feared events that increase postoperative morbidity and mortality. Our cohort of patients demonstrated no intraoperative complications associated with LTCBDE. Although absent within the literature, one feared complication associated with accessing the CBD through a transcystic route is avulsion of the cystic duct at its junction with the CBD. Additionally, injury to the common bile duct is also a feared complication and has only been reported in one case series (*n* = 1, < 0.01%).

Postoperative complications and adverse events were absent within our cohort of patients. This is lower than previously reported data, ranging from 4.6% to 16.7% [7–9, 12, 14]. This is likely due to our relatively low number of patients. Unlike treatment modalities that access the CBD from a retrograde fashion, the risk of postprocedural pancreatitis is much lower via the transcystic route, owing to the very low risk of cannulating the pancreatic duct. Although not completely zero, this risk may be as high as 5.5% and is more likely to occur in an emergent setting than in an elective setting [12, 14, 24]. 

The mean operative time of our cohort was 178 min, which includes the time spent performing the laparoscopic cholecystectomy, performing and interpreting the intraoperative cholangiogram, and performing the LTCBDE. This is consistent with operative times reported in the literature, which range from 150 to 168 min [13, 25, 26]. A benefit to the LTCBDE is the ability to complete both the cholecystectomy and the CBD exploration in one procedure, in a reasonable amount of time. Other treatment modalities, such as balloon enteroscopy, TG-ERCP, and EDGE procedures, often require multiple trips to the procedure suite or operating room, exposing patients to multiple anesthetic events, lengthy operative/procedural times, and increased overall hospital length of stay, which translates into unnecessary costs to the healthcare system.

The hospital and postoperative length of stay varied significantly based on the admitting service. We demonstrated a significantly shorter hospital and postoperative LOS when patients were admitted directly to a surgical service instead of a medicine service (Table 4). This difference has been demonstrated in studies comparing single-stage laparoscopic cholecystectomy with laparoscopic CBD exploration and two-stage procedures with preoperative ERCP followed by laparoscopic cholecystectomy during the same hospital admission [26, 27]. This difference in LOS has implications for patient outcomes, hospital throughput, and overall cost of the hospital stay.

General Surgeons caring for patients suffering from biliary disease should be comfortable with performing and interpreting intraoperative cholangiograms as part of their training. Furthermore, the American Board of Surgery now requires surgical trainees to complete the Fundamentals of Endoscopic Surgery (FES) curriculum, demonstrating competency in basic endoscopic techniques [28]. The presence of altered foregut anatomy, such as RYGB, does not inherently change the operative technique for cholecystectomy but represents a safe and effective option for these patients in the hands of surgeons who should be adequately equipped to care for them. Our data support this management plan and should be considered as a first-line option for any patient presenting with choledocholithiasis who has a gallbladder in situ.

### Study Limitations

We identify that there are many limitations inherent to retrospective, descriptive, cohort studies concerning the accuracy of information within medical records. Additionally, selection bias is a concern; however, at our institution, any patient with choledocholithiasis who has undergone a previous RYGB operation is referred to our minimally invasive and bariatric surgery division for treatment, which is the same division that performs the LTCBDE, thereby minimizing the risk of this bias. The risk of recall bias increases when discussing postoperative details with patients during telephone follow-up, particularly for patients who are further removed from the index procedure. This study aimed to illustrate the safety and efficacy of the LTCBDE. We intentionally excluded a comparison group due to our small sample size, which would make comparative analyses challenging. In the future, prospective randomized studies will provide additional data comparing the efficacy and safety of the LTCBDE and other interventions for treating choledocholithiasis in patients with RYGB anatomy.

## Conclusions

Our study demonstrates that LTCBDE is both safe and effective for treating choledocholithiasis in patients with RYGB anatomy. We also showed a shorter LOS for patients admitted to a surgical service, suggesting that patients seen acutely for choledocholithiasis who have a gallbladder would benefit from being admitted to a surgical service upon presentation to the hospital. Performing LTCBDE at the time of cholecystectomy upon presentation to the hospital can prevent the need for multiple procedures with the cumulative risk of postoperative morbidities and shorten the overall hospital stay.

## Data Availability

No datasets were generated or analysed during the current study.
